# Beyond oncology: Selinexor’s journey into anti-inflammatory treatment and long-term management

**DOI:** 10.3389/fimmu.2024.1398927

**Published:** 2024-05-10

**Authors:** Dan Li, Hong Fang, Rong Zhang, Qian Xie, Yang Yang, Lin Chen

**Affiliations:** ^1^ Respiratory Medicine Department, Wuhou District People's Hospital, Chengdu, China; ^2^ Department of Pulmonary and Critical Care Medicine, Sichuan Provincial People's Hospital, School of Medicine, University of Electronic Science and Technology of China, Chengdu, China; ^3^ Department of Pulmonary and Critical Care Medicine, Mayo Clinic, MN, United States

**Keywords:** Selinexor, oncology, inflammatory diseases, nuclear export inhibition, therapeutic mechanisms

## Abstract

Selinexor, a selective inhibitor of nuclear export (SINE), is gaining recognition beyond oncology for its potential in anti-inflammatory therapy. This review elucidates Selinexor’s dual action, highlighting its anti-tumor efficacy in various cancers including hematologic malignancies and solid tumors, and its promising anti-inflammatory effects. In cancer treatment, Selinexor has demonstrated benefits as monotherapy and in combination with other therapeutics, particularly in drug-resistant cases. Its role in enhancing the effectiveness of bone marrow transplants has also been noted. Importantly, the drug’s impact on key inflammatory pathways provides a new avenue for the management of conditions like sepsis, viral infections including COVID-19, and chronic inflammatory diseases such as Duchenne Muscular Dystrophy and Parkinson’s Disease. The review emphasizes the criticality of managing Selinexor’s side effects through diligent dose optimization and patient monitoring. Given the complexities of its broader applications, extensive research is called upon to validate Selinexor’s long-term safety and effectiveness, with a keen focus on its integration into clinical practice for a diverse spectrum of disorders.

## 1 Introduction

Selinexor, also known as KPT-330, is an oral small molecule drug that Selectively Inhibits Nuclear Export (SINE). It specifically inhibits exportin 1 (XPO1), which exports various proteins (including tumor suppressor proteins and growth regulators) from the nucleus to the cytoplasm (1). By inhibiting XPO1, Selinexor effectively traps these proteins in the nucleus, leading to the reactivation of tumor suppressor functions and induction of apoptosis in cancer cells. Selinexor’s mechanism of action distinguishes it from other oncology therapies, making it a viable clinical option for treating various tumor types, such as multiple myeloma and diffuse large B-cell lymphoma (1). Additionally, Selinexor has shown potential therapeutic applications beyond its initial approval for hematological malignancies, including breast cancer (2), lung adenocarcinoma (3), and gastric cancer (4), by inducing cell cycle arrest and promoting apoptosis.

However, Selinexor has emerged as a pivotal agent in the treatment of certain oncologic conditions, particularly demonstrating efficacy in scenarios where traditional therapies have failed. It is important to recognize that Selinexor is not intended as a first-line treatment option, nor is it recommended for direct comparison with standard treatment modalities. Its utility is specifically recognized in the treatment of drug-resistant or treatment-refractory cases, offering a novel avenue for patients who have exhausted other therapeutic options. This distinct role underscores the importance of understanding Selinexor’s unique mechanism of action, its therapeutic potential, and the need for precise patient selection criteria to optimize treatment outcomes.

But it is encouraging that Selinexor has demonstrated not only anti-tumor effects but also anti-inflammatory effects and protection against other inflammatory diseases, such as COVID-19 (5), sepsis (6), and Duchenne muscular dystrophy (DMD) (7). The applications of Selinexor will not be limited to oncology, and there may be broader areas that can be explored. The role of Selinexor will be explored in detail.

## 2 Selinexor in anti-cancer treatment

### 2.1 Selinexor in hematologic malignancies

Selinexor has shown effectiveness against blood cancers by inhibiting tumor growth and inducing the death of cancer cells, with a better safety profile for healthy cells compared to older treatments. Studies indicate its success as both a solo treatment and in combination with other therapies across various blood cancer types.

In significant research, the STORM trial investigated Selinexor in patients with advanced myeloma who had undergone numerous treatments, finding that approximately 26% experienced a reduction in cancer severity (8). The Boston trial examined the combination of Selinexor with bortezomib and dexamethasone in 402 patients, revealing that this regimen extended progression-free survival to nearly 14 months, offering notable benefits particularly to older individuals and those with renal issues (9).

The SADAL study evaluated Selinexor in individuals with advanced Large B-Cell Lymphoma after 2-5 prior treatments, reporting a 28% response rate and an average survival of 9 months with manageable side effects (10). An additional investigation into various Non-Hodgkin’s Lymphomas (NHL) in 70 patients showed a 31% response rate, supporting Selinexor’s role in treating relapsed or refractory cases (11).

Regarding other blood cancer types, a Phase I clinical trial combining Selinexor with chemotherapy for relapsed/refractory Acute Myeloid Leukemia (AML) reported a 70% overall response rate and a 50% complete remission rate (12). Another AML study with Selinexor, cytarabine, and idarubicin showed a 47.6% response rate and a median complete remission duration of 34 days, highlighting variable outcomes in such AML treatments (13). In chronic lymphocytic leukemia (CLL), Selinexor has been found to boost the effects of chemotherapeutic agents like Fludarabine and Bendamustine, or sustain the impact of PI3Kδ inhibitors, effectively overcoming resistance to single-agent therapies and preserving the drugs’ ability to kill tumor cells (14).

### 2.2 Selinexor in non-hematologic malignancies

Selinexor is broadening its horizons from blood cancers to include treatments for solid tumors, showcasing its versatility in oncology. This expansion reflects an active pursuit to uncover its full potential beyond hematologic applications, emphasizing the necessity for comprehensive clinical evaluations across various cancer types.

For advanced and metastatic malignancies, Selinexor has demonstrated a promising safety and efficacy profile. A Phase 1 study underscored its tolerability in patients with a range of advanced cancers, suggesting a potential for broader applicability (15).

In the realm of soft tissue sarcoma (STS), Selinexor’s combination with doxorubicin has yielded partial remission in 21% of patients, and stable disease in 63% (16, 17). This finding is significant, particularly for a diverse and rare cancer like STS, highlighting Selinexor’s capacity to fill therapeutic gaps where targeted options are limited.

Gynecological cancers, too, have seen potential benefits from Selinexor, with a Phase I study indicating partial or complete remission in patients treated with a combination of Selinexor, paclitaxel, and carboplatin (18). This promising result opens up new avenues for treatment in ovarian and endometrial cancers.

Research has broadened Selinexor’s application to encompass a variety of solid tumors, addressing complex cases such as salivary gland tumors, recurrent glioblastoma, metastatic triple-negative breast cancer, and castration-resistant prostate cancer (19–22). Although the outcomes of these studies vary, they collectively indicate potential clinical benefits of Selinexor across diverse solid tumors. Notably, the integration of Selinexor with radiotherapy has been shown to enhance apoptosis and reduce proliferation in colorectal cancer cell lines and xenograft tumor models, compared to either treatment alone. This synergistic effect has led to decreased tumor sizes and improved responses to radiation (23).

This exploration into non-hematologic malignancies with Selinexor represents a significant stride in cancer treatment, hinting at a versatile and effective option for a range of solid tumors. The ongoing challenge is to fine-tune treatment protocols and deepen understanding of its optimal use, particularly in combination therapies and specific patient demographics, to maximize Selinexor’s therapeutic advantage.

### 2.3 Summary of Selinexor in anti-tumor treatment

Selinexor, typically not used as a standalone therapy in antitumor regimens, excels when combined with other treatments, especially for blood cancers. This strategy offers renewed hope for patients who have exhausted other therapeutic options. The drug’s ability to enhance the efficacy of combination therapy regimens is marked by its capacity to attenuate the harsh effects of chemotherapy and maintain cancer cell sensitivity to ongoing treatments (24–26). Moreover, the integration of Selinexor into treatment strategies not only improves outcomes for resistant cases but also aids in preparing patients for bone marrow or stem cell transplants. By reducing cancer burden pre-transplant, Selinexor increases the likelihood of successful chemotherapy preparatory regimens, broadening the eligibility for these life-extending procedures (27–29).

While Selinexor introduces significant therapeutic benefits, its administration is not without challenges. A primary concern is hematological toxicities such as thrombocytopenia, which affects approximately 54% of treated patients, necessitating close monitoring and potential dose adjustments to mitigate severe bleeding risks (30). Gastrointestinal side effects are prevalent, with nausea and vomiting reported frequently. These effects can substantially impact patient quality of life and adherence to the therapy regime. Effective management strategies often involve supportive care measures, including the use of antiemetics and dietary adjustments to help patients better tolerate treatment (30). Concerns about hepatotoxicity are underscored by instances of elevated ALT levels, signaling potential liver injury. Though relatively rare, regular monitoring of liver function is essential for detecting any hepatic injury early, allowing for timely medical intervention (30). Additionally, Selinexor treatment has been associated with a heightened risk of infections, particularly upper respiratory tract infections, observed in about 17.8% of patients in clinical trials. This necessitates rigorous monitoring and preemptive management strategies to mitigate the risk and manage any infections promptly (30).

The use of biomarkers plays a critical role in monitoring Selinexor’s efficacy and managing resistance. Studies such as the BOSTON and STORM trials have uncovered a three-gene signature (WNT10A, DUSP1, and ETV7) that predicts Selinexor’s efficacy in treating multiple myeloma, in terms of both depth and duration of response (31). ABCC4, or ATP-binding cassette subfamily C member 4, has emerged as a significant biomarker for Selinexor sensitivity in multiple myeloma, with varying expression levels correlating with the drug’s effectiveness. This suggests ABCC4’s potential as a novel indicator of drug response, highlighted by weighted gene co-expression network analysis demonstrating its predictive value (32). Further, CRISPR-Cas9 screening has identified ASB8 as a critical element enhancing Selinexor sensitivity in various cancer types through the modulation of XPO1 proteasomal degradation. Additionally, the TGFβ-SMAD4 pathway has been identified as a significant factor in resistance to multiple myeloma, suggesting its potential as a biomarker for predicting therapeutic outcomes and devising strategies to overcome resistance (33). Monitoring the activity or expression of XPO1 and NF-kB, which are directly tied to Selinexor’s mechanism of action, can provide valuable insights into treatment effectiveness. These biomarkers are instrumental in adjusting therapeutic approaches to maximize patient benefits while minimizing adverse effects (34–36).

In summary, Selinexor’s role in overcoming chemotherapy resistance and facilitating bone marrow transplants underscores its value in treating blood cancers. This approach could significantly improve the chances of a cure for some patients.

## 3 Introduction to Selinexor and inflammation

The rationale for exploring Selinexor’s role in inflammatory pathways stems from its unique mechanism of action. By inhibiting XPO1, Selinexor effectively traps crucial regulatory proteins within the nucleus, thereby impeding their normal function in the cytoplasm. This action leads to the modulation of several cellular pathways involved in cell survival, inflammation, and immune responses. The nuclear retention of these proteins can result in the downregulation of pro-inflammatory cytokines and the modulation of other key components of inflammatory pathways.

This broad mechanism suggests a potential utility for Selinexor beyond oncology, targeting inflammatory and autoimmune diseases where dysregulation of cytokine signaling plays a significant role. The anti-inflammatory effects of Selinexor have been observed in various models of disease, providing a promising outlook for its application in treating chronic and acute inflammatory conditions.

### 3.1 Mechanism of action of Selinexor in inflammation

#### 3.1.1 Focusing on proteins involved in inflammation

Selinexor targets several key pathways central to inflammation, notably the NF-κB and STAT3 signaling pathways. Under typical conditions, NF-κB is confined in the cytoplasm bound to IκB proteins. In response to inflammatory stimuli, IκB degrades, allowing NF-κB to enter the nucleus and activate genes that escalate the inflammatory response. Selinexor intervenes by stabilizing IκBα, thus hindering the nuclear translocation of NF-κB. This prevention reduces the transcription of pro-inflammatory cytokines and mediators, critical components that perpetuate inflammation. The suppression of these mediators is crucial for controlling inflammatory processes across various conditions (6).

Building on this mechanism, Selinexor also modulates the STAT3 signaling pathway, which plays a pivotal role in mediating inflammation. By retaining STAT3 in the nucleus, Selinexor limits its capability to activate downstream genes responsible for the inflammatory response. This containment reduces the overall inflammatory activity, providing therapeutic benefits especially in diseases where STAT3 is overly active (37).

Selinexor’s influence on immune regulation is primarily achieved through its action on NF-κB and STAT3, critical transcription factors involved in immune cell activation. By retaining these factors in the nucleus, Selinexor prevents them from promoting the expression of genes that drive the inflammatory and immune responses. This results in a reduction of immune cell activation and inflammatory signaling, making it beneficial for treating conditions with excessive immune activation (6).

#### 3.1.2 Changes in cytokine profiles due to Selinexor treatment

Cytokines, small proteins crucial for cell signaling, play significant roles in the immune response during inflammation. Key cytokines like tumor necrosis factor-alpha (TNF-α), interleukins (IL-1β, IL-6), and interferons (IFNs) drive inflammatory responses, and their dysregulation can exacerbate disease severity and progression. Selinexor’s ability to modulate cytokine production largely stems from its inhibition of nuclear export. By blocking the nuclear export of transcription factors such as NF-κB and STAT3, Selinexor prevents them from activating genes responsible for pro-inflammatory cytokine production. This suppression leads to a notable reduction in cytokine levels, directly impacting the inflammatory process (6).

Clinically, Selinexor’s effect on cytokine levels has profound implications. It has been shown to significantly reduce concentrations of key pro-inflammatory cytokines, thereby alleviating symptoms and lessening disease severity. For instance, in diseases like rheumatoid arthritis or inflammatory bowel disease, where cytokine overproduction leads to tissue damage, Selinexor’s ability to modulate cytokine production can prevent tissue damage and reduce symptoms (38).

Furthermore, by altering cytokine profiles, Selinexor offers a new therapeutic approach for conditions inadequately managed by existing medications. This includes diminishing chronic disease flare-ups, reducing reliance on steroids or other immunosuppressants, and potentially improving patient quality of life. Additionally, the altered localization of signaling molecules through Selinexor’s action affects downstream signaling pathways essential for immune cell development and function, notably reducing the proliferation and differentiation of pro-inflammatory T cells crucial in autoimmune and inflammatory diseases.

#### 3.1.3 Specific effects on T cells

Selinexor modulates T cell function primarily through the indirect effects of inhibiting nuclear export, which is crucial since T cells are central to both cell-mediated immunity and the modulation of other immune responses through cytokine production. By altering the localization and activity of key transcription factors such as NF-κB and STAT3, which are vital for T cell activation and proliferation, Selinexor prevents these factors from triggering the gene expression necessary for T cell responses. This retention within the nucleus significantly curtails the proliferation and activation of T cells, particularly those implicated in inflammatory responses (38).

Furthermore, T cells are prolific producers of cytokines like interleukin-2 (IL-2), interferon-gamma (IFN-γ), and tumor necrosis factor-alpha (TNF-α), which drive inflammation. By reducing the availability of transcription factors necessary for cytokine gene activation, Selinexor effectively diminishes cytokine production (39). This reduction is crucial for alleviating the inflammatory response, offering therapeutic benefits in diseases characterized by cytokine storms or persistent inflammation.

The suppression of inappropriate immune responses by Selinexor, especially in autoimmune diseases where T cells target self-antigens, alleviates symptoms associated with these conditions. By dampening T cell activation and reducing inflammatory cytokine levels, Selinexor aids in managing inflammatory diseases and contributes to maintaining immune homeostasis. These actions underscore Selinexor’s potential to modulate the immune system in ways fundamentally different from traditional immunosuppressive or immunostimulatory therapies, providing a novel approach to treating conditions where immune regulation is beneficial, such as autoimmune disorders and chronic inflammation.

Furthermore, Selinexor’s ability to prevent the creation of neutrophil extracellular traps (NETs) while maintaining neutrophil function provides strategic benefits in the treatment of illnesses defined by high NET activity (40). It affects cell death mechanisms, such as PANoptosis (pyroptosis, apoptosis, and necroptosis), by controlling the nuclear export of ADAR1-p150, highlighting its potential for treating cancer and inflammatory illnesses by modulating cell death pathways (41).

**Figure 1:**
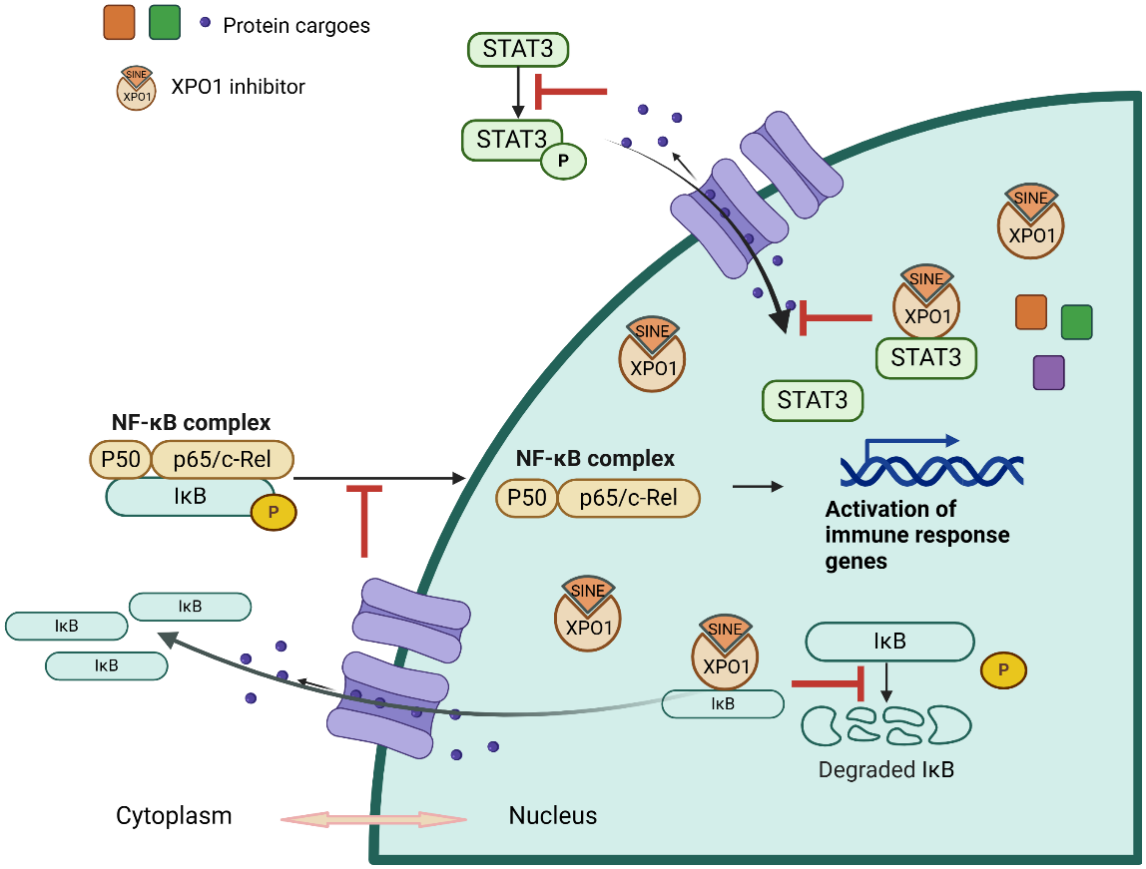
Illustration of Selinexor’s Mechanism of Action in Modulating Inflammatory Pathways. This diagram depicts the mechanism of action of Selinexor in inhibiting inflammation. It shows the stabilization of IκBα, which prevents the translocation of NF-κB to the nucleus and the subsequent reduction in pro-inflammatory cytokine transcription. The image outlines Selinexor’s effect on various components of the inflammatory pathway, highlighting its role in downregulating cytokine production and modulating immune responses.

The therapeutic scope of Selinexor in anti-inflammatory therapy is complicated, encompassing nuclear output inhibition, cytokine production control, modulation of critical signaling pathways, and antiviral actions, as illustrated in **Figure 1**. These features make Selinexor and other SINE compounds intriguing candidates for the treatment of chronic inflammatory disorders and viral infections, paving the way for further study into their broad therapeutic applications.

### 3.2 Clinical implications of Selinexor’s anti-inflammatory actions

#### 3.2.1 Sepsis

Clinical studies have established Selinexor’s efficacy in modulating key inflammatory pathways in sepsis. In animal models, treatment with Selinexor has been observed to significantly improve survival rates. Specifically, it reduces lung damage caused by lipopolysaccharide (LPS) and decreases the accumulation of inflammatory cells in the peritoneal area. These effects are linked to reduced levels of pro-inflammatory cytokines such as TNF-α, IL-6, and HMGB1 (6). Selinexor’s mechanism of action in sepsis involves the inhibition of crucial inflammatory pathways, notably NF-kB and MAPK p38, which are integral to the inflammatory cascade in sepsis. This inhibition is significant as it directly affects the pathways that exacerbate sepsis, providing a targeted approach to reducing acute inflammatory reactions (6).

#### 3.2.2 Antiviral applications

Selinexor and Verdinexor, members of the SINE chemical family, demonstrate potent antiviral properties by inhibiting the nuclear export of viral proteins crucial for assembly and replication. In animal models, including ferrets in COVID-19 studies, Selinexor has effectively reduced viral loads and mitigated inflammatory damage, indicating its role in managing severe viral infections through immune modulation and cytokine storm reduction (37). Verdinexor disrupts the lifecycle of Respiratory Syncytial Virus (RSV) by sequestering RSV M proteins in the cell nucleus, inhibiting replication of both A and B strains. This action simultaneously enhances p53 activity and reduces XPO1 levels, offering a strategic advantage against RSV (42).

In the context of COVID-19, Selinexor blocks the nuclear export of essential viral proteins such as ORF3b, ORF9b, and the nucleocapsid N protein, crucial for SARS-CoV-2 replication. It also reduces the cell surface presence of the ACE-2 receptor, limiting viral entry. Furthermore, Selinexor modulates immune responses by controlling the release of inflammatory cytokines, potentially alleviating cytokine storms commonly associated with severe COVID-19 cases through the inhibition of NF-kB pathways and direct effects on STAT3 and IL-6 transcription (37). Conversely, research by Rahman et al. suggests that pretreatment with Selinexor may enhance coronavirus replication, including in SARS-CoV-2 and mouse hepatitis virus (MHV), illustrating the complex effects of Selinexor on viral replication and inflammation, and underscoring the necessity for precise administration timing (40, 43).

These findings reveal the dual potential of Selinexor and KPT-335 in combating viral infections, showcasing their ability to control viral growth and inflammation. Despite promising initial results, the complexities and some contradictory findings underscore the need for further research to fully understand their therapeutic potential beyond cancer treatment and to integrate them into broader therapeutic frameworks.

#### 3.2.3 Chronic inflammatory diseases

Selinexor, along with other SINE compounds such as KPT-8602, exhibits potential in treating both acute and chronic inflammatory diseases, spanning conditions like Duchenne Muscular Dystrophy (DMD), Parkinson’s Disease (PD), Calcific Aortic Valve Disease (CAVD), and pulmonary fibrosis. These compounds inhibit key inflammatory pathways, offering new therapeutic strategies across a spectrum of diseases.

In chronic conditions like DMD, Selinexor and KPT-8602 have shown promise by modulating inflammatory cytokines and pathways, improving muscle function, and slowing disease progression. Specifically, KPT-8602 not only enhances muscular architecture but also reduces serum levels of biomarkers associated with bone and muscle turnover, fostering conditions conducive to muscle healing and renewal (7). This dual approach targets both the structural damage and inflammatory underpinnings of DMD, making KPT-8602 a valuable therapeutic option. For PD, these compounds protect dopaminergic neurons by mitigating neuroinflammation, thereby potentially delaying the progression of the disease. The inhibition of critical pathways such as NF-kB and the NLRP3 inflammasome underscores their capacity to protect against neuronal degeneration, a hallmark of PD (44). In the realm of CAVD, Selinexor has demonstrated the ability to modulate inflammatory responses that contribute to pathological calcification, suggesting a role in managing diseases characterized by such calcifications (42). Recent insights into Selinexor’s impact on pulmonary fibrosis reveal its effectiveness through the inhibition of the XPO1 protein, influencing GBP5/NLRP3 inflammasome signaling pathways crucial in the inflammatory responses associated with fibrosis. This action suggests that Selinexor could be instrumental in treating conditions marked by widespread inflammation and fibrotic transformations (45).

Overall, the exploration of SINE compounds in treating benign diseases has garnered significant attention due to promising results that suggest a potential to control, if not decelerate, the progression of these conditions (42, 44). The broad applicability of SINE compounds in diverse inflammatory and autoimmune conditions supports continued research into their comprehensive anti-inflammatory effects across various diseases.

## 4 Limitations and future directions

As Selinexor’s role in treating both cancerous and non-cancerous conditions continues to be explored, it is evident that while the drug offers promising results in managing inflammation, there are significant gaps and limitations in our understanding that necessitate further investigation. One primary concern is Selinexor’s specificity as an XPO1 inhibitor, which influences a broad spectrum of proteins and can lead to unintended consequences, including severe side effects. This broad activity presents challenges, especially in chronic conditions where long-term treatment is required, as balancing efficacy and safety becomes critical (46, 47). The need for optimal dosing to achieve the desired anti-inflammatory effects without compromising safety is a significant hurdle, with higher doses potentially increasing the risk of adverse effects.

Additionally, while it is recognized that Selinexor modulates several key inflammatory pathways, the precise mechanisms by which it impacts specific conditions remain poorly understood. This incomplete mechanistic insight hampers the development of targeted therapies that could enhance Selinexor’s efficacy while minimizing side effects.

Looking ahead, future research should focus on developing targeted delivery systems that can localize Selinexor’s action to specific tissues or organs. This strategy would potentially reduce systemic side effects and enhance efficacy in localized inflammatory conditions, such as inflammatory bowel disease or rheumatoid arthritis. Exploring combination therapies that include Selinexor and other anti-inflammatory agents may also improve therapeutic effects and possibly reduce the necessary dosages, which could be tailored to specific pathways active in various inflammatory diseases.

Longitudinal studies are crucial for assessing the long-term effects and safety of Selinexor in chronic inflammation. Such studies would provide a more comprehensive understanding of its benefits and risks. Further research into identifying biomarkers that predict responses to Selinexor could improve patient selection and treatment monitoring, facilitating personalized treatment approaches that optimize therapeutic outcomes while minimizing adverse effects.

Moreover, expanding research to explore Selinexor’s effects in non-cancerous inflammatory diseases could uncover additional therapeutic uses and provide new insights into its anti-inflammatory properties, potentially opening new avenues for treatment across a broader range of inflammatory disorders. This ongoing research and the development of new clinical strategies are essential for fully realizing the potential of Selinexor in the management of both malignant and benign diseases, ensuring that treatments are both effective and safe for long-term use.

## 5 Conclusion

Selinexor is distinguished by its unique nuclear inhibition, exhibiting extensive effects. By trapping tumor suppressor proteins within the cell nucleus, Selinexor induces apoptosis in cancer cells, proving effective against various types of tumors, including hematologic malignancies and solid tumors. This positions it as a key therapeutic approach in oncology, especially valuable in cases resistant to conventional treatments.

Similarly, Selinexor demonstrates robust anti-inflammatory action through its key mechanism of XPO1 inhibition, which suppresses the activity of regulatory factors such as NF-kB and STAT3. This action effectively modulates immune responses and inflammatory pathways, enhancing its utility in treating both acute and chronic inflammatory diseases such as sepsis, DMD, and PD. This dual functionality not only broadens its therapeutic applications beyond oncology but also highlights its potential to manage a diverse array of diseases.

However, the transition of Selinexor from an anti-cancer to an anti-inflammatory agent has unveiled both its versatility and the complexities associated with its broader applications. Ongoing research is crucial in further unraveling its full potential, aiming to harness its capabilities while addressing any associated risks more effectively. The extensive clinical trials and continuous research efforts are essential to ensure the long-term safety and efficacy of Selinexor, optimizing its use in clinical practice.

As research advances, Selinexor continues to stand out not only for its significant therapeutic implications but also as a paradigm shift in the management of complex disorders. Its ability to concurrently address oncological and inflammatory diseases underscores its potential as a versatile therapeutic tool. This highlights the importance of continued investigations into its mechanisms and therapeutic applications to fully realize and utilize its extensive benefits.

## Author contributions

DL: Writing – original draft, Investigation. HF: Writing – original draft, Data curation. RZ: Writing – review & editing, Resources. QX: Writing – review & editing. YY: Writing – original draft. LC: Writing – review & editing, Supervision, Conceptualization.

## Glossary

**Table:**

SINE	Selectively Inhibits Nuclear Export
XPO1	Exportin 1
COVID-19	Coronavirus Disease 2019
DMD	Duchenne muscular dystrophy
DDR	DNA damage response
STORM	Selinexor Treatment with Refractory Myeloma
BOSTON	Bortezomib, Selinexor, and Dexamethasone in Patients with Multiple Myeloma
SADAL	Selinexor in patients with relapsed or refractory diffuse large B-cell lymphom
NHL	Non-Hodgkin’s Lymphoma
AML	Acute Myeloid Leukemia
CLL	Chronic lymphocytic leukemia
STS	Soft Tissue Sarcoma
NK	Natural Killer
HLA-E	Human Leukocyte Antigen E
NF-kB	Nuclear Factor Kappa-Light-Chain-Enhancer of Activated B cells
IκBα	Inhibitor of NF-kB alpha
STAT3	Signal Transducer and Activator of Transcription 3
NETs	Neutrophil Extracellular Traps
ADAR1-p150	Adenosine Deaminase Acting on RNA 1, p150 isoform
LPS	lipopolysaccharide
RSV	respiratory syncytial virus
HMGB1	High Mobility Group Box 1
MHV	mouse hepatitis virus
FLAG-Ida	Fludarabine, Cytarabine, Granulocyte colony-stimulating factor (G-CSF), and Idarubicin
ALT	Alanine Transaminase
CRISPR-Cas9	Clustered Regularly Interspaced Short Palindromic Repeats” and “CRISPR associated protein 9
ASB8	Ankyrin Repeat and SOCS Box Containing 8
TGFβ-SMAD4	Transforming Growth Factor Beta-SMAD Family Member 4 Pathway
WNT10A	Wnt Family Member 10A
DUSP1	Dual Specificity Phosphatase 1
ETV7	ETS Variant Transcription Factor 7
ABCC4	ATP-binding cassette subfamily C member 4
WGCNA	weighted gene co-expression network analysis
NFATc1	Nuclear Factor of Activated T Cells 1
PD	Parkinson’s Disease
NLRP3	NLR Family Pyrin Domain Containing 3
CAVD	calcific aortic valve disease
